# Missed on X-ray, Found on CT: A Retrospective Study on the Diagnostic Yield and Clinical Consequences of Occult Posterior Malleolus Fractures in Tibial Shaft Fractures

**DOI:** 10.7759/cureus.94260

**Published:** 2025-10-10

**Authors:** Hariprasath Kanesan, Zain Choudhary, Sachin Singal, Mahesh Kanesan, Ronald Hang-Kin Nam, Niranj Ganeshan Radhamony, Mohamed Hamadto

**Affiliations:** 1 Orthopedics and Trauma, Royal Stoke University Hospital, Stoke, GBR; 2 Surgery, Royal Shrewsbury Hospital, Shrewsbury, GBR

**Keywords:** ct-imaging, distal tibia fracture, orif distal tibia fibula, posterior malleolus fracture, tibial shaft fracture, tibia nail, x-ray images

## Abstract

Background

Posterior malleolus fractures (PMFs) are common in distal tibial shaft fractures yet are frequently occult on plain radiographs. Accurate preoperative characterization on CT may improve surgical planning and functional recovery.

Methods

We conducted a retrospective cohort study of consecutive adults (≥18 years) with mid- or distal tibial shaft fractures treated at a major trauma center (January 2022-December 2024). Demographics, imaging, fracture characteristics, management, and outcomes were abstracted. PMF detection was compared across radiography, CT, and intraoperative findings. Predictors of a posterior malleolus (PM) fragment being missed on radiography were evaluated with multivariable logistic regression. Among operatively treated cases, the effect of fixation strategy on postoperative full weight‑bearing (FWB) was analyzed with a logistic model including fixation, PM status, and their interaction; pairwise, covariate‑adjusted contrasts (emmeans with Tukey correction) compared strategies. An exploratory random forest provided permutation‑importance rankings.

Findings

PMFs were present in 147/387 fractures (38.0%), more often in women (60.5%), closed injuries (76.9%), and distal fractures (98.0%). Radiography identified 116/147 PMFs (78.9%), and CT 120/147 (81.6%); 29/147 (19.7%) fragments were missed on radiographs but detected on CT or intraoperatively. Missed fragments were most frequent in undisplaced and intermediate‑sized (33-50%) fragments; CT identified all posteromedial and >50% fragments. In adjusted analyses, fracture morphology was the principal correlate of radiographic omission: oblique patterns were less likely to be missed (adjusted odds ratio 0.18, 95% CI 0.04-0.60), whereas other demographic and injury variables were not significant. Among operatively managed fractures, intramedullary nailing was associated with the highest likelihood of achieving FWB and was statistically superior to plate and external fixation in pairwise, covariate‑adjusted comparisons; the relative ranking of fixation methods did not differ by PM involvement. Neither PMF location (posteromedial, posterolateral, or undisplaced) nor fragment size independently predicted FWB, infection, or mal‑/non‑union.

Conclusions

Approximately two in five distal tibial shaft fractures harbour a PM fragment, and nearly one in five PMFs are occult on initial radiographs, particularly when undisplaced or of intermediate size. CT modestly increases detection and ensures complete characterisation of posteromedial and large fragments, supporting a low threshold for pre‑operative CT in distal tibial fractures.

## Introduction

Recent studies investigated how posterior malleolus fractures (PMFs) and tibial shaft fractures are often underdiagnosed on standard radiographs and require computed tomography (CT) imaging preoperatively in order to improve surgical approach for surgical fracture fixation [1]. With roughly 187 in 100000 individuals sustaining ankle fractures each year, the National Health Service (NHS) faces considerable strain; timely and accurate investigation and diagnosis can help prevent fracture-related complications [2]. 

Recent evidence points to the shortcomings of standard X-rays in identifying PMF [3], especially in cases involving low-energy distal tibial shaft injuries. Late detection secondary to inappropriate imaging modalities can lead to an estimated complication rate of up to 25% [4]. Therefore, it is essential for surgeons to have appropriate imaging in a timely manner to allow the best fixation method to be selected, which has been shown to play a significant role in determining clinical outcomes [5]. Routine preoperative CT imaging can enhance the accuracy of diagnosis in identifying PMF and prevent delay of fracture management [6]. As a result of this, it is important to evaluate the diagnostic value of routine preoperative CT imaging in detecting PMF, especially in low-energy distal tibial shaft fractures. Institutions with standardized CT imaging protocols have reported improvements in identifying intra-articular injuries not visible on X-rays, yet such protocols are not consistently applied in clinical practice. 

Despite mounting evidence supporting the diagnostic superiority of CT over plain radiographs, there remains a lack of consensus on the routine use of preoperative CT imaging in distal tibial shaft fractures. Institutions with standardized CT imaging protocols have reported improvements in identifying intra-articular injuries not visible on X-rays [7], yet such protocols are inconsistently applied in clinical practice. There remains a lack of standardized guidelines on the routine use of CT imaging in the context of tibial shaft fractures, resulting in variable clinical practices [7]. 

Furthermore, the degree of PMF displacement and the selection of surgical approach have not been well established, leaving a significant gap in evidence-based management of these injuries. This rationale underpins the present retrospective cohort study, which analyzed clinical and radiological data from a well-defined patient cohort who sustained tibial shaft fractures with or without associated PMF at a major trauma center. Data was extracted from medical records and imaging archives from the years 2022 to 2024, focusing on fracture displacement, surgical intervention, and preoperative imaging modalities. This design allows for a robust evaluation of real-world clinical outcomes and the identification of factors associated with optimal surgical decision-making. 

Study objectives

This study aimed to evaluate the diagnostic value of CT imaging compared with radiographs and intra-operative inspection for detecting PM fragments in distal tibial shaft fractures and to evaluate the degree of PM fragment displacement and its influence on the choice of surgical approach.

## Materials and methods

Ethical approval

Ethical approval for this study was waived by Royal Stoke University Hospital (submission number: CA09825) following protocol review. The project was classified as a service evaluation and therefore did not require formal ethical approval. 

Inclusion criteria

This retrospective study included all consecutive patients aged 18 years and older who presented to the hospital with a mid- or distal tibial shaft fracture at the study centre over a 36-month period (January 1, 2022, to December 31, 2024). The study was conducted at Royal Stoke University Hospital, University Hospitals of North Midlands NHS Trust, Stoke-on-Trent, United Kingdom. The study centre is a major trauma centre serving a catchment population of approximately three million people. Patients under 18 years old and with ankle fractures were excluded. 

Imaging protocol and assessment of posterior malleolus fractures

Radiographs were obtained according to the standard trauma protocol (anteroposterior, lateral, and mortise views). CT selection criteria and acquisition CT was performed selectively at the discretion of the admitting trauma and orthopedic team and approved by the on-call radiology registrar, reflecting variation in local practice. CT examinations were eligible for inclusion in CT-based analyses if they included the distal tibia and ankle joint and were performed between emergency department presentation and definitive fixation. CTs performed solely after definitive fixation were not used for PM detection or morphology. Standard trauma CT protocols were used (≤1 mm slice thickness with multiplanar reconstruction in axial, coronal, and sagittal planes). All eligible CTs within the study period were included consecutively; no sampling was performed.

Observer assessment and blinding images were initially reported by a trained radiology registrar and subsequently cross-checked and verified by a consultant radiologist as per routine workflow; the final, consultant-verified report was taken as the clinical interpretation. Radiologists had access to clinical notes (including clerking notes and working diagnoses) and other imaging when reporting. Posterior malleolus fragment size and morphology were assessed by the treating orthopedic registrar or consultant as part of preoperative planning, using multiplanar CT images where available. Fragment size was estimated as the proportion of the tibial plafond involved, and anatomical location was described as posterolateral, posteromedial, or central. Multiple observers contributed across the study period. No inter-observer reproducibility testing was performed. Readers were not blinded to clinical information or other imaging for the purposes of this study, and no blinding was used during data analysis.

Data collection

Patients were retrospectively identified from the trauma and orthopedics admission spreadsheet. Patient demographics, American Society of Anaesthesiologists (ASA) grade, location of fracture, mechanism of injury, isolation of injury, association with PMF, identification of PMFs on X-ray, CT, and intraoperatively, method of management, surgical approach, weight-bearing status postoperatively, union, and presence of infection were collected from the electronic health records. All patients were followed up for a minimum of six weeks, except for patients who were repatriated or died. Data is obtained and permitted to answer clinical questions by the trust’s approval for service evaluation of current practice (register number: CA09825). All data was handled in accordance with the United Kingdom Caldicott principles.

Clinical outcome definitions

Full weight‑bearing (FWB) was defined as surgeon-permitted, unaided FWB on the operated limb, usually assessed at the six-week postoperative visit. Union was defined radiographically as bridging callus across at least three of four cortices, supported clinically by the absence of pain on weight-bearing

Handling of missing data

No participant was excluded from the cohort due to missing data. Descriptive analyses were conducted on an available-case basis with denominators stated. Inferential analyses were performed on complete cases with respect to variables included in each model (i.e., participants with missing values for any model variable were excluded from that model only). Variables with more than 30% missingness were not included as covariates in multivariable models, and analyses in which a key exposure or outcome had more than 30% missingness were not reported. The extracted dataset was of good quality, and no analysis was excluded on this basis. Given this approach, no imputation methods were used. 

Statistical analysis 

All analyses were conducted in R version 4.4.2 (R Foundation for Statistical Computing, Vienna, Austria). Categorical variables are summarised as counts and percentages, and continuous variables as means (SD) or medians (IQR), as appropriate. Group comparisons used Pearson’s χ² test or Fisher’s exact test for categorical data and t tests for continuous data.

Prevalence and detection route of PM fragments by modality (radiograph, CT, and intra‑operative inspection) was tabulated, and the proportion missed on plain radiographs but detected on CT or intra‑operatively was reported.

Predictors of missed PM fragments on radiography were assessed using multivariable logistic regression. Candidate predictors were prespecified and included age (per 10‑year increase), sex, injury mechanism (high vs low energy), open versus closed fracture, tibial fracture site (mid‑shaft vs distal), and fracture pattern (oblique, spiral, transverse; reference: comminuted). Adjusted odds ratios (aORs) with 95% CIs are reported. 

For postoperative FWB, the comparative effectiveness of fixation strategies was evaluated among operatively managed cases only (screws, intramedullary nail, plate, and external fixation). We fitted a multivariable logistic model including fixation strategy, PM status, and their interaction (Fixation×PM), adjusted for the same covariates (age, sex, mechanism, open/closed, tibial site, fracture pattern). The primary inference for “superiority” among fixation methods used model‑based, covariate‑adjusted pairwise comparisons of estimated marginal means (emmeans), with Tukey correction for multiple testing. Pairwise results are presented as odds ratios (obtained by exponentiating differences on the logit scale) with 95% CIs and adjusted p-values. For clinical interpretability, adjusted predicted probabilities of FWB (with 95% CIs) were also reported for each fixation-PM combination.

As an exploratory analysis of predictor importance for missed PM on radiographs, we fitted a classification random forest (1000 trees) with permutation importance; mean decrease in accuracy was used to rank variables. This machine‑learning analysis was used to corroborate signals from the regression models and is not the basis for formal inference.

Analyses were performed on complete cases. Statistical significance was defined as two‑sided p<0.05. Emphasis was placed on effect sizes and precision rather than sole reliance on p-values.

## Results

Baseline characteristics

A total of 387 consecutive tibial‑shaft fractures were included; 147 (38.0%) had an associated PMF (Table 1). Mean age was 50.9 years (SD 19.4), and 184 (47.5%) patients were women. Compared with those without a PMF, patients with a PMF were more frequently female (60.5% vs 39.6%; p<0.001), sustained distal rather than mid‑shaft fractures (98.0% vs 73.3%; p<0.001), and were more likely to have closed injuries (76.9% vs 53.3%; p<0.001) and isolated presentations (90.5% vs 80.4%; p=0.009). Injury severity (ASA class) did not differ between groups (both means 2.0; p=0.915). Low‑energy mechanisms predominated in the PMF cohort (74.1% vs 50.4%; p<0.001). Fracture morphology is summarised in Table 2; comminuted fractures were most frequent overall.

**Table 1:** Demographics (with p-values) PM: posterior malleolus; ASA: American Society of Anaesthesiologists

Characteristic	All Tibial Fractures	No PM	PM	p-value
Number (n)	387	240	147	NA
Age (mean (SD))	50.93 (19.36)	50.20 (19.72)	52.14 (18.75)	0.333
Female (%)	184 (47.5%)	95 (39.6%)	89 (60.5%)	<0.001
ASA (mean (SD))	2.05 (0.84)	2.05 (0.88)	2.04 (0.79)	0.915
Closed (%)	241 (62.3%)	128 (53.3%)	113 (76.9%)	<0.001
Isolated Injury (%)	326 (84.2%)	193 (80.4%)	133 (90.5%)	0.009
High impact (%)	157 (40.6%)	119 (49.6%)	38 (25.9%)	<0.001
Distal tibial fracture (%)	320 (82.7%)	176 (73.3%)	144 (98.0%)	<0.001
Mid-shaft tibial fracture (%)	67 (17.3%)	64 (26.7%)	3 (2.0%)	<0.001

**Table 2:** Fracture pattern distribution by sex and age group

Group	Total	Comminuted	Oblique	Spiral	Transverse
All	387	211 (54.5%)	95 (24.6%)	45 (11.6%)	36 (9.3%)
M	203	125 (61.6%)	35 (17.2%)	23 (11.3%)	20 (9.9%)
F	184	86 (46.7%)	60 (32.6%)	22 (12.0%)	16 (8.7%)
<30	58	35 (60.3%)	13 (22.4%)	3 (5.2%)	7 (12.1%)
30–59	200	110 (55.0%)	45 (22.5%)	25 (12.5%)	20 (10.0%)
60+	129	66 (51.2%)	37 (28.7%)	17 (13.2%)	9 (7.0%)

Detecting PM fragments

Detection routes are shown in Tables 3, 4. Plain radiography identified 116 of 147 PM fragments (78.9%); CT detected 120 (81.6%), and intraoperative inspection documented a fragment in 26 cases (17.7%). Twenty‑nine fragments (19.7%) were missed on plain radiography but found on CT or at operation. By displacement (Table 3), the proportions missed on radiography were 25.0% (4/16) for posteromedial, 21.4% (15/70) for undisplaced, and 17.5% (10/57) for posterolateral fragments. By size (Table 4), the missed‑on‑radiography rate was highest for intermediate‑sized fragments occupying 33-50% of the plafond (10/30, 33.3%), lower for small fragments ≤33% (18/96, 18.8%), and zero for fragments >50% (0/19). Posteromedial fragments were detected by CT in all cases (16/16), whereas posterolateral and undisplaced fragments were also documented intraoperatively in 19.3% and 12.9% of cases, respectively. Fragments >50% were always seen on both radiography and CT. Intraoperative documentation occurred in 12.5% of small (≤33%) and 46.7% of intermediate (33-50%) fragments; for intermediate fragments, this was in addition to CT detection (100%).

**Table 3:** Detection route by location of PM fragment displacement PM: posterior malleolus

Detect	Total	Posteromedial	Posterolateral	Undisplaced
Total	147	16	57	70
XR	116 (78.9%)	11 (68.8%)	46 (80.7%)	55 (78.6%)
CT	120 (81.6%)	16 (100.0%)	54 (94.7%)	47 (67.1%)
Intra-op	26 (17.7%)	5 (31.2%)	11 (19.3%)	9 (12.9%)
XR -ve; CT/OP +ve	29 (19.7%)	4 (25.0%)	10 (17.5%)	15 (21.4%)

**Table 4:** Detection route by size of PM fragment PM: posterior malleolus

Detect	Total	≤33%	33–50%	>50%
Total	147	96	30	19
XR	116 (78.9%)	76 (79.2%)	20 (66.7%)	19 (100.0%)
CT	120 (81.6%)	70 (72.9%)	30 (100.0%)	19 (100.0%)
Intra-op	26 (17.7%)	12 (12.5%)	14 (46.7%)	0 (0.0%)
Missed XR	29 (19.7%)	18 (18.8%)	10 (33.3%)	0 (0.0%)

Clinical predictors of missed posterior malleolus fragments on radiography

In multivariable logistic regression (Table 5), fracture morphology was the principal determinant of a missed fragment on radiography. An oblique pattern was independently associated with a substantially lower risk of omission (adjusted odds ratio (aOR) of 0.18, 95% CI 0.04-0.60; p=0.011).

**Table 5:** Multivariable logistic regression predicting missed posterior malleolus fragments † NE = not estimable owing to complete/quasi separation.

Variable	Adjusted OR (95 % CI)	p-value
Age (per 10-year increase)	0.90 (0.67–1.18)	0.445
Male sex	2.70 (0.99–7.65)	0.055
Low-energy mechanism	1.52 (0.45–5.82)	0.516
Open fracture	2.01 (0.68–5.85)	0.200
Poly-trauma	1.48 (0.30–6.78)	0.619
Mid-shaft tibial site †	NE	—
Oblique fracture pattern	0.18 (0.04–0.60)	0.011
Spiral fracture pattern	4.17 (0.81–22.29)	0.084
Transverse fracture pattern	0.40 (0.02–2.66)	0.421

A spiral pattern showed a non‑significant increase in risk (aOR 4.17, 0.81-22.29; p=0.084), and a transverse pattern had no discernible effect (aOR 0.40, 0.02-2.66; p=0.421). Age, sex, energy of injury, fracture openness, and poly‑trauma were not significantly associated with radiographic detection. The effect of a mid‑shaft tibial site could not be estimated because of quasi‑/complete separation and is reported as not estimable.

**Figure 1:**
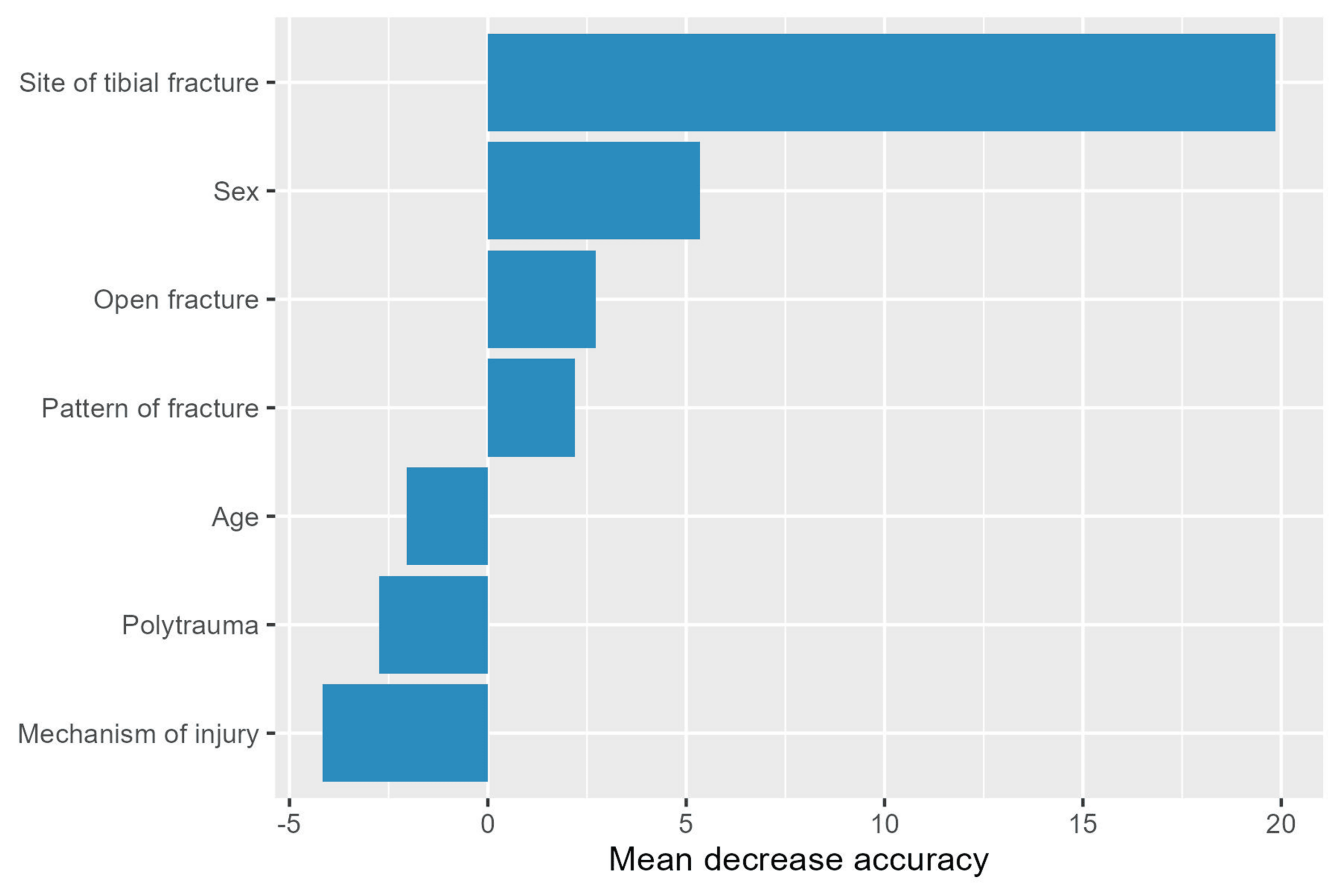
Random forest variable importance for predicting missed fragments on radiography

In an exploratory machine‑learning analysis using a random forest (1000 trees) with permutation importance (Figure 1), the tibial site ranked highest (mean decrease in accuracy (MDA) 19.50), followed by fracture openness (3.49), sex (3.47), and overall fracture pattern (2.04). Age (−1.78), poly‑trauma (−5.07), and injury mechanism (−5.62) did not improve out‑of‑bag predictive accuracy (negative MDA indicates no meaningful contribution). These rankings corroborate the regression signal, namely, a strong anatomical contribution (tibial site) and a pattern effect, while highlighting that the tibial‑site effect, although influential for classification, could not be stably quantified as an odds ratio in the parametric model.

Association of PM fragment displacement or size with outcome

Among patients with PMF, neither fragment displacement location (posteromedial, posterolateral, or undisplaced) nor fragment size was independently associated with failure to achieve FWB, infection, or mal‑/non‑union after surgical fixation (Tables 6-8). Intermediate‑sized fragments (33-50%) showed a borderline increase in the odds of FWB (aOR 4.51, 95% CI 1.04-22.47; p=0.051). No displacement or size category was significantly associated with infection (Table 7) or mal‑/non‑union (Firth logistic regression; Table 8). Other demographic and injury‑related factors were not significantly associated with these outcomes after adjustment.

**Table 6:** Adjusted odds ratios for full weight-bearing

Predictor	OR (95% CI)	p-value
Posteromedial displacement	1.25 (0.33–4.62)	0.739
Undisplaced fragment	1.59 (0.63–4.13)	0.334
Size ≤33 %	1.10 (0.29–4.79)	0.893
Size 33–50 %	4.51 (1.04–22.47)	0.051
Age (per 10 y)	1.07 (0.85–1.36)	0.558
Male sex	0.94 (0.39–2.27)	0.887
Low-energy mechanism	1.59 (0.60–4.41)	0.356
Open fracture	2.03 (0.75–5.56)	0.164
Mid-shaft location	3.38 (0.21–107.05)	0.409
Oblique pattern	0.45 (0.18–1.07)	0.075
Spiral pattern	0.16 (0.01–1.18)	0.118
Transverse pattern	0.22 (0.03–1.16)	0.101

**Table 7:** Adjusted odds ratios for infection

Label	OR (95 % CI)	p-value
Undisplaced fragment	2.55 (0.35–28.22)	0.358
Size ≤33 % (ref >33 %)	0.65 (0.08–7.22)	0.691
Age (per 10 y)	1.32 (0.75–2.72)	0.346
Male sex	0.67 (0.05–5.39)	0.716
Low-energy mechanism	0.57 (0.05–7.87)	0.640
Open fracture	2.62 (0.39–17.80)	0.307
Oblique pattern	0.80 (0.07–6.77)	0.836
Other pattern	1.88 (0.16–16.71)	0.576

**Table 8:** Adjusted odds ratios for malunion/nonunion (Firth logistic regression)

Label	OR (95 % CI)	p-value
Undisplaced fragment	0.29 (0.00–4.53)	0.407
Size ≤33 % (ref >33 %)	0.75 (0.05–9.49)	0.808
Age (per 10 y)	1.29 (0.66–3.42)	0.456
Male sex	0.48 (0.00–6.51)	0.603
Low-energy mechanism	1.52 (0.06–230.69)	0.802
Open fracture	1.66 (0.10–24.25)	0.700
Oblique pattern	0.34 (0.00–5.48)	0.474
Other pattern	1.22 (0.01–27.09)	0.908

Effect of fixation strategy and PM involvement on achieving full weight-bearing postoperatively

Pairwise, covariate‑adjusted comparisons showed that intramedullary nailing was superior to plate and external fixation for achieving FWB (overall: nail vs plate odds ratio (OR) 12.24, 95% CI 4.24-35.37; nail vs. external fixation OR 12.57, 3.62-43.66; both Tukey‑adjusted p<0.001; Table 9). Screw fixation did not differ significantly from any other method (all p>0.44), and plate and external fixation were not different (p=1.00). Findings were consistent within strata of PM involvement (No PM: nail vs plate OR 20.09, 3.91-103.27, p<0.001; nail vs external fixation OR 15.28, 2.84-82.33, p<0.001; PM present: nail vs plate OR 7.46, 2.01-27.62, p<0.001; nail vs external fixation OR 10.34, 1.77-60.35, p=0.004; Table 10). The fixation‑by‑PM interaction was not significant (p=0.26), indicating that the relative ranking of fixation methods did not differ by PM status. The presence of a PM fragment itself was not an independent predictor of outcome after adjustment. The global effect of fixation was highly significant (likelihood‑ratio p<0.0001), underscoring fixation strategy as the principal determinant of postoperative FWB in this cohort.

**Table 9:** Pairwise adjusted comparisons among fixation methods (overall; Tukey-adjusted)

Fixation Strategies	Odds ratio (95% CI)	P-value
Screws - Nail	0.37 (0.03–4.66)	0.740
Screws - Plate	4.47 (0.34–59.54)	0.446
Screws - (Ex-fix)	4.59 (0.30–69.65)	0.474
Nail - Plate	12.24 (4.24–35.37)	<0.001
Nail - (Ex-fix)	12.57 (3.62–43.66)	<0.001
Plate - (Ex-fix)	1.03 (0.26–4.04)	1.000

**Table 10:** Pairwise adjusted comparisons among fixation methods stratified by posterior malleolus status (Tukey-adjusted)

PM	Fixation Strategies	Odds ratio (95% CI)	P-value
No PM	Screws - Nail	0.44 (0.01–17.20)	0.939
No PM	Screws - Plate	8.82 (0.18–435.42)	0.478
No PM	Screws - (Ex-fix)	6.71 (0.13–339.55)	0.598
No PM	Nail - Plate	20.09 (3.91–103.27)	<0.001
No PM	Nail - (Ex-fix)	15.28 (2.84–82.33)	<0.001
No PM	Plate - (Ex-fix)	0.76 (0.09–6.43)	0.988
PM	Screws - Nail	0.30 (0.01–9.89)	0.816
PM	Screws - Plate	2.27 (0.08–64.26)	0.923
PM	Screws - (Ex-fix)	3.14 (0.08–118.32)	0.849
PM	Nail - Plate	7.46 (2.01–27.62)	<0.001
PM	Nail - (Ex-fix)	10.34 (1.77–60.35)	0.004
PM	Plate - (Ex-fix)	1.39 (0.29–6.58)	0.950

Interpretation 

PMFs accompanied nearly two in five distal tibial shaft fractures, occurring more often in women and after low‑energy, closed injuries. Around one in five PMFs was not visible on initial radiographs; intermediate‑sized (33-50%) and undisplaced fragments were most frequently missed, and small numbers suggest posteromedial fragments can also be overlooked. CT modestly increased overall detection and identified all posteromedial and larger fragments; nevertheless, intraoperative documentation remained common, particularly for intermediate‑sized fragments. On multivariable analysis, fracture morphology was the principal correlate of radiographic omission: oblique patterns were less likely to be missed, whereas spiral patterns showed a non‑significant tendency to be overlooked; demographic and injury‑energy variables were uninformative. An exploratory random‑forest analysis corroborated the importance of tibial site and fracture pattern for classification. Neither fragment location (posteromedial, posterolateral, or undisplaced) nor fragment size independently predicted failure of FWB, infection, or mal‑/non‑union after fixation. In comparative effectiveness analyses among operatively treated fractures, intramedullary nailing yielded higher odds of FWB than plate and external fixation, with similar ranking irrespective of PM involvement; screw constructs performed comparably to nails. These findings support a low threshold for preoperative CT in distal tibial fractures to avoid unrecognized PM injury, particularly when undisplaced or intermediate‑sized fragments are possible, while emphasizing that, when adequately treated, fragment displacement alone does not appear to compromise early function or union.

## Discussion

Our study highlights the high prevalence and diagnostic complexity of PMFs in distal tibial shaft fractures as well as the critical role of CT imaging in their detection. Among 387 tibial shaft fractures, PMFs were present in 38%, most frequently in the context of closed, low-energy injuries in women. Despite the widespread use of plain radiographs, almost 20% of PM fragments were not recognized until CT imaging or intraoperative examination, particularly those that were undisplaced or of intermediate size (33-50%).

These findings strengthen previous research showing the limitations of plain radiographs, particularly in detecting subtle or posteromedial fragments. Hou et al. demonstrated that CT imaging is superior for characterizing both the presence and morphology of PMFs, particularly when displacement is subtle or posteromedial in location [5]. CT imaging provided a higher detection rate (81.6% vs. 78.9%) and ensured complete identification of posteromedial fragments, which are known to be radiographically occult in many cases. Our results support the routine use of preoperative CT in all distal tibial shaft fractures, particularly when oblique fracture patterns or medium-sized fragments are suspected, both of which were independent predictors of missed fragments on plain films. These findings align with other studies advocating for the use of CT in assessing suspected PMF in distal tibial injuries [7-11].

Our results support routine preoperative CT in distal tibial shaft fractures, given that almost one in five PM fragments were occult on radiographs. Missed fragments were most commonly undisplaced or of intermediate size (33-50%), and small numbers suggest posteromedial fragments can also be overlooked. Fracture morphology influenced detectability, with oblique patterns less likely to be missed and spiral morphology showing a non‑significant tendency towards omission. These findings align with prior work advocating CT to fully characterize PM involvement in distal tibial injuries.

The clinical relevance of identifying PMFs preoperatively lies in ensuring accurate surgical planning and avoiding complications such as ankle instability, malreduction, or other poor functional outcomes if displaced fragments are undetected [12,13]. However, in contrast to previous reports that emphasized the role of fragment displacement or size in guiding fixation strategy, our findings suggest a more nuanced relationship. Neither PMF displacement position (posteromedial, posterolateral, or undisplaced) nor fragment size was independently associated with increased risk of poor postoperative outcomes, such as failure to achieve FWB, infection, or malunion. Medium-sized fragments (33-50%) showed a non-significant trend toward improved functional outcome (aOR 4.5, p = 0.051), but this did not reach statistical significance.

Moreover, fixation strategy had a strong effect on outcome; intramedullary nailing was associated with the most favorable weight‑bearing results, and this advantage did not differ by PM involvement (Fixation×PM interaction p=0.26). Fragment displacement, position, and size were not independently associated with outcomes.

These findings challenge earlier surgical algorithms that emphasized fixation of large (>25%) or displaced PM fragments as critical for functional recovery [14,15]. More recent studies have questioned the adequacy of using size alone to determine fixation need, particularly in the setting of complex tibial fractures [9]. Our results support this updated perspective and suggest that with modern surgical techniques, outcomes are more closely tied to overall fracture characteristics and soft-tissue considerations than to PMF size or orientation alone.

In summary, this study supports a shift in clinical practice toward routine preoperative CT scanning for distal tibial shaft fractures to improve diagnostic accuracy and surgical planning. At the same time, it suggests that fragment displacement and size are not independently predictive of adverse outcomes and therefore should not override other established criteria when formulating a fixation strategy. These insights may inform the development of evidence-based imaging and surgical protocols aimed at improving both diagnostic precision and long-term functional results.

Strengths

This retrospective study offers important strengths that enhance its validity and clinical impact. Firstly, it addresses a clinically relevant question about the detection and management of PMFs in distal tibial fractures, an area of ongoing debate. The study includes a data set of 387 tibial‑shaft fractures over a three-year period, providing a robust dataset that captures real-world patterns across a high-volume major trauma center.

The study is strengthened by advanced statistical approaches, including multivariable logistic regression, covariate-adjusted pairwise comparisons using estimated marginal means, and an exploratory machine-learning random forest analysis to corroborate findings and identify key predictors of missed diagnosis.

Also, the work of this study goes beyond imaging detection. It includes clinically meaningful functional outcomes, such as FWB, infection, and union status, directly linking diagnostic and surgical decisions to patient recovery. Together, these features strengthen the reliability, interpretability, and practical applicability of the results, providing actionable insights for improving preoperative planning and standardizing imaging protocols.

Limitations

This study is a retrospective analysis conducted at a single trauma center. As a retrospective study, it may be subject to selection and information biases. Additionally, being single-center, the findings may have limited generalizability to other institutions or practice settings. To enhance the robustness and external validity of the results, future research could consider combining data from multiple centers.

With non-universal CT use, there is a potential for verification bias, which may have influenced detection rates of PMFs and could have led to under- or overestimation of their true prevalence. It is important to recognize that the observed prevalence reflects the local threshold for ordering CT, rather than the true prevalence in the broader fracture population. This is due to imaging being performed selectively by the admitting team and the radiology service. Such variability in practice can complicate comparisons between centers that use different imaging thresholds. 

Furthermore, the lack of detail on imaging protocols and measurement reproducibility could have introduced variability in fragment classification. Without uniform imaging criteria and blinded, repeat assessments, reproducibility across settings cannot be assured. The lack of reproducibility testing for imaging assessment is important to consider when recommending routine CT scans, as the incremental benefit observed here may not be reproducible in centers with different practices.

Additionally, confounding by indication is possible, as the choice of fixation was made by the operating surgeon rather than by randomization. This decision was often based on fracture pattern, soft tissue status, and patient factors. These same factors may also influence other measured outcomes, such as weight-bearing status, infection, or union. This makes it difficult to fully separate the effect of fixation technique from underlying characteristics despite statistical adjustment.

Importantly, outcomes were only assessed over a short period of time, with most patients followed up for a minimum of six weeks. While this captures early functional milestones such as FWB, it does not provide insights into longer-term outcomes, such as chronic pain, post-traumatic arthritis, or late complications, which are highly relevant in evaluating the clinical significance of missed or occult fragments.

Taken together, these limitations emphasize the need for well-designed, prospective multi-center studies that use standardized imaging protocols, reproducible measurement methods, and clearly defined outcome criteria. Such studies would help confirm our findings, reduce bias, and provide stronger evidence to guide imaging strategies and management decisions for distal tibial shaft fractures.

## Conclusions

This study shows that PMFs are common in distal tibial shaft fractures and that plain radiography frequently fails to detect them, particularly when the fragments are undisplaced or of intermediate size. CT modestly improves detection and reliably identifies posteromedial and large fragments, suggesting that maintaining a low threshold for preoperative CT can aid surgical planning, especially in distal fractures with suspicious morphology. Fragment location and size did not independently predict early functional or union outcomes in this cohort, although the study may not have been powered to exclude smaller effects. Taken together, these findings support maintaining a low threshold for CT in distal tibial shaft fractures, particularly when radiographs are inconclusive or when identifying a PM fragment could influence surgical planning. These results highlight the need for prospective, multicenter studies with standardized imaging and longer follow-up to confirm these results and refine practice guidelines.
